# Placental expression of pituitary hormones is an ancestral feature of therian mammals

**DOI:** 10.1186/2041-9139-2-16

**Published:** 2011-08-19

**Authors:** Brandon R Menzies, Andrew J Pask, Marilyn B Renfree

**Affiliations:** 1 Leibniz Institute for Zoo and Wildlife Research, Alfred-Kowalke-Str 17, 10315, Berlin, Germany; 2 Department of Molecular and Cell Biology, University of Connecticut Storrs, CN, USA; 3 Department of Zoology, The University of Melbourne, 3010, Victoria, Australia

## Abstract

**Background:**

The placenta is essential for supplying nutrients and gases to the developing mammalian young before birth. While all mammals have a functional placenta, only in therian mammals (marsupials and eutherians) does the placenta closely appose or invade the uterine endometrium. The eutherian placenta secretes hormones that are structurally and functionally similar to pituitary growth hormone (GH), prolactin (PRL) and luteinizing hormone (LH). Marsupial and eutherian mammals diverged from a common ancestor approximately 125 to 148 million years ago and developed distinct reproductive strategies. As in eutherians, marsupials rely on a short-lived but functional placenta for embryogenesis.

**Results:**

We characterized pituitary GH, GH-R, IGF-2, PRL and LHβ in a macropodid marsupial, the tammar wallaby, *Macropus eugenii*. These genes were expressed in the tammar placenta during the last third of gestation when most fetal growth occurs and active organogenesis is initiated. The mRNA of key growth genes GH, GH-R, IGF-2 and PRL were expressed during late pregnancy. We found significant up-regulation of GH, GH-R and IGF-2 after the start of the rapid growth phase of organogenesis which suggests that the placental growth hormones regulate the rapid phase of fetal growth.

**Conclusions:**

This is the first demonstration of the existence of pituitary hormones in the marsupial placenta. Placental expression of these pituitary hormones has clearly been conserved in marsupials as in eutherian mammals, suggesting an ancestral origin of the evolution of placental expression and a critical function of these hormones in growth and development of all therian mammals.

## Background

Growth hormone (GH) is a fundamental regulator of normal post-natal growth in mammals and is also important to maintain lipid, carbohydrate, nitrogen, and mineral metabolism [1]. The GH gene has changed little during the evolution of mammals [2]. However, in higher primates and ruminant artiodactyls, the gene has undergone duplications, followed by rapid, independent spurts of evolution with the result that sequences from these species are highly dissimilar to all others [3]. Four GH-like genes exist in humans and include the three chorionic somatomammotropins (A, B and L) and one GH variant (GH-V or placental GH) all of which are produced exclusively by the placenta [4]. These genes share a high degree of sequence and structure similarity, consisting of five exons and four introns located in tandem on the long arm of chromosome 17, and each encode a mature protein of approximately 190 to 200 amino acids [4]. Interestingly, exon 3 of the GH receptor (GH-R), which is responsible for extra-cellular binding and transmission of the GH signal, is not present in marsupials or monotremes [5]. While this does not affect post-natal growth, the exon 3 sequences of eutherian mammals with placental variants of GH and PRL are much more variable than those without placental hormone variants. Human GH-V has recently been shown to play an important role in trophoblast invasion into the endometrium by stimulating its receptor GH-R [6].

In sheep, there are two allelic variations of the growth hormone gene. The GH-1 allele contains one copy of pituitary GH while the GH-2 allele contains two tandem copies of the GH gene designated *GH2-N *and *GH2-Z *[7]. *GH2-N *codes for pituitary GH while *GH2-Z *contains three amino acid substitutions and is expressed only in the sheep placenta. While the GH-2 allele appears to be more frequent in sheep populations than that of GH-1, animals that are homozygous or heterozygous for either allele still produce pituitary GH in the placenta and there are no growth differences between fetuses of varying allelic composition [7]. While the goat has a similar makeup of GH genes, having shared a common ancestor with the sheep approximately 5 million years ago, there is also evidence for duplicate GH genes in the chevrotain, red deer, giraffe and hippopotamus [2]. However, it is unknown whether these individual duplications are homologous to the other groups.

Eutherian mammals such as rodents and cows have evolved specific prolactin variants that are expressed in the placenta. A total of 21 such genes exist in rodents and 8 in the cow [8]. Within the Rodentia, many of these variants are assumed to be orthologues. However, the cow and rodent variants are not orthologous, demonstrating the independent duplication and divergence of these sequences.

Placental expression of pituitary hormone variants including GH, PRL and LH-β (of which human chorionic gonadotropin (HCG) provides the first signal of an ensuing pregnancy from the human conceptus), likely originate from tissue specific expression by the conceptus or the placenta, followed by gene duplication of the original gene for a specific function. If this is correct, then the placentas of a diverse array of mammals would be expected to produce the pituitary form of these hormones for generalized functions in pregnancy. Marsupials provide an ideal model in which to examine the evolution of these genes. Marsupials have a fully functional but short-lived placenta that elaborates at least some hormones [9]. However, the highly altricial young that are delivered after a very short pregnancy complete their development over an extended lactational period, either in a pouch or a nest [10]. Eutherians and marsupials last shared a common ancestor 125 to 147 million years ago [11,12] so it is possible to determine whether placental expression and gene duplication was a common feature of the ancestral mammal or was a more recent evolutionary event that occurred only in the eutherian lineage.

The first marsupial pituitary GH sequence was isolated in the brushtail possum, *Trichosurus vulpecula *[13] and shares considerable sequence identity with pig and horse GHs (species in which GH is highly conserved) with approximately 87% protein identity. This suggests a conserved rate of GH evolution in marsupials similar to that described for the majority of mammals [2,13]. The possum prolactin and red kangaroo LH-β sequences have also been isolated and share considerable protein identity with other mammals [14,15].

Pituitary GH sequences have been cloned from a broad group of mammals. However, comparisons with placental GH, PRL and LH have mainly been examined in cow, sheep, rat, mouse, human and other primates [8]. Thus, expression of these important hormones appears to be a general feature of eutherians and, as in humans, may be providing diverse functions in metabolism and pregnancy recognition. No marsupials have been examined as yet, and almost nothing is known of the conservation and expression of pituitary hormones in any non-eutherian mammal. This study therefore investigates the sequence composition and expression of the pituitary hormones GH, PRL, and LH-β in the placenta of a marsupial the tammar wallaby *Macropus eugenii*. We have also quantified expression in the trilaminar placenta over the last third of pregnancy when active organogenesis occurs.

## Results

### Isolation and expression of pituitary genes in the tammar placenta

**Figure 1:**
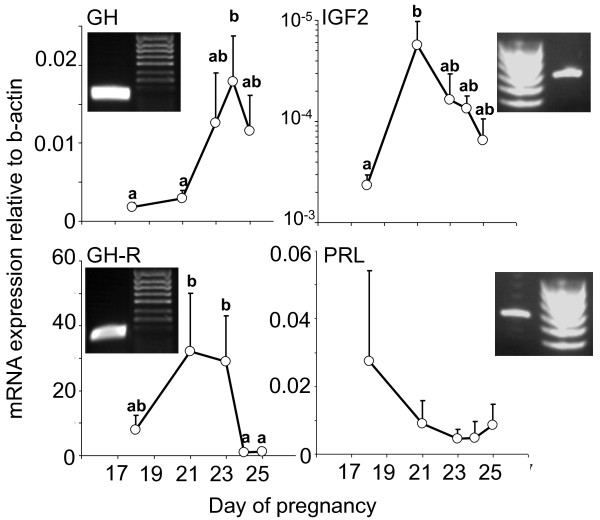
**Quantitative expression of *GH, GH-R, IGF-2 *and *PRL *in the trilaminar yolksac placenta**. Relative mRNA expression of the growth genes *GH, GH-R *and *IGF-2 *all increased significantly compared to *β-actin *after shell coat rupture in the tammar placenta. This period of pregnancy is characterized by rapid fetal growth and development prior to birth of the altricial young (n = 3 to -6 per stage; letters indicate significant differences *P *< 0.05; there were no significant changesin prolactin (PRL) expression over this period; **GH, growth hormone; GH-R, growth hormone receptor; IGF-2, insulin-like growth factor-2)**.

First we isolated the pituitary sequence of GH to establish whether there was any sequence variation indicative of placental variants. Pituitary GH was amplified and sequenced in the adult tammar (n = 2; both male). It had considerable protein identity with the brushtail possum (99%; Table 1) and other mammals. The same primer set (Table 2) was used to amplify and sequence this gene from the placenta at day 23 of gestation (675 base pairs; Figure 1). The resulting sequence spanned numerous introns and covered the whole open reading frame (ORF). There were no sequence differences between wallaby pituitary and placental GH. Next, we analyzed placental tissue for the GH receptor. Full length GH-R cDNA was previously cloned from the livers of adult tammars and their pouch young [5]. There was no variation in the *GH-R *mRNA sequence in the bilaminar (BYS) and trilaminar (TYS) placenta compared to that of the liver *GH-R *mRNA. There was strong expression of *GH *and *GH-R *in both the tri- and bilaminar placenta from day 18 to 25 of pregnancy (189 base pairs; Figure 1).

**Table 1:** Protein alignment values for the tammar GH, PRL, and LH- β genes compared to other marsupial and eutherian mammals

Common name	Species name	GH homology (%)	PRL homology (%)	LH-β homology (%)
Brushtail possum	*Trichosurus vulpecula*	99	97	97
Grey short-tailed opossum	*Monodelphis domestica*	96	93	94
Pig	*Sus scrofa*	85	85	80
Horse	*Equus caballus*	85	85	73
Rabbit	*Oryctolagus cuniculus*	82	84	83
Human	*Homo sapiens*	65^a^	85	66^a^

^a^variability associated with evolution of placental variants.

**Table 2:** Genes, primers, annealing temperatures (Tm) and number of PCR cycles used to amplify specific genes

Gene	F primer	R primer	Tm	PCR cycles	Accession ID
*GH*	GGACCTGGAAGAAGGGATTC	CACAGCTGCTTTCCACAAAA	60°C	30-35	EU918392
*GH-R*	CCCTGGCAAGTTCAAATGAT	GGCATTCTTTCCACTCTTCG	60°C	30-35	EU682376
*PRL*	ACCACCAACTCCTTCTGGTG	GGAGCAAATTATAAAATGCA	58°C	30-35	FJ434245
*LH-β*	AGATACCAGGAGCTCACAGT	GAATTTGGGGGATGGAAAAG	58°C	30-35	FJ434244
*IGF-2*	CCTTTGTGGTGGGGAACTGGT	GGATGGGGTCTTCGCTGGGCA	60°C	30-35	AM993150

**Figure 2:**
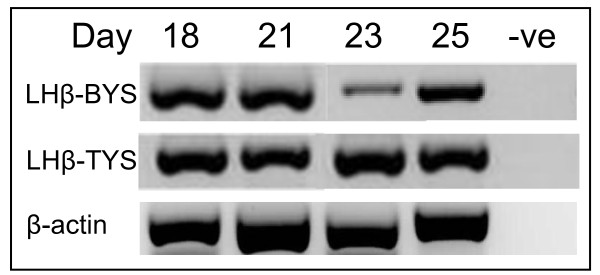
**LH-β expression in the tammar wallaby placenta**. mRNA gene expression in placental tissue from days 18 to 25 of pregnancy in the tammar wallaby. The isolation and expression of this pituitary gene in the marsupial placenta suggests that orthologues of these genes may be found in the placentas of all mammals. (BYS: bilaminar yolk sac; La: DNA Ladder; TYS: trilaminar yolk sac; -ve: negative control).

The mRNAs for *PRL *and *LH-β *were also first isolated from the pituitary in order to compare these sequences with placental isolates. Both of these sequences shared considerable protein identity with that of the brushtail possum (97% each; Table 1; Additional file 1, Figure S1) and other mammals. The inferred PRL protein had many conserved structural features including 6 cysteine residues which form 3 disulfide bridges that are important for the 3-dimensional structure of the protein, as well as 15 amino acids which are necessary for receptor binding [16]. Similarly, the inferred LH-β glycoprotein contained several conserved elements including cysteine residues necessary for binding to the LH-α chain (Additional file 2, Figure S2). Placental *PRL *and *LH-β *mRNA were expressed from day 18 to 25 of pregnancy in both tri- and bilaminar yolk sac placenta (652 and 509 base pairs, respectively; Figure 1; Figure 2). The placental mRNA sequences for *PRL *and *LH-β *encompassed most of the ORFs of these genes similar to GH and spanned multiple introns. Placental *PRL *and *LH-β *isolates were identical to the pituitary isolates.

### Quantitative expression of GH, GH-R and IGF-2

**Figure 3:**
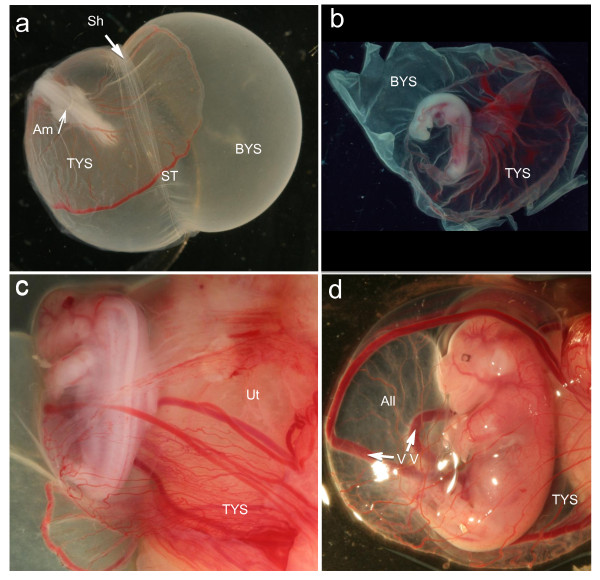
**Growth and development of the tammar embryo and fetus**. **(a) **At day 18, the conceptus emerges form the ruptured shell coat (Sh) so that the two regions of the placenta can make a close attachment to the uterine epithelium. The right side of the embryo is already coat-free, while the embryo itself is still within the shell coat. **(b) **The d18 embryo free of the fetal membranes, clearly distinguishing between the vascular and non-vascular regions of the yolk sac (BYS: bilaminar yolk sac; TYS trilaminar yolk sac; ST sinus terminalis). **(c) **Fetus at day 23 of pregnancy, showing the increase in the vascular region and the close attachment of the placenta to the uterine epithelium (Ut). The vitelline vessels are prominent. **(d) **Full term fetus at day 25 of pregnancy, about a day before birth. The allantois (All) is large but held within the folds of the yolk sac, which has become highly vascular. The fetus has well developed fore limbs ready for the climb to the pouch and the tongue is typically protruded (Am: amniopore).

Expression of the growth genes GH, GH-R and IGF-2 were all low during the period of shell coat rupture (day 18; Figure 1). After the loss of the shell coat (Figure 3), attachment and interdigitation of the trophoblast with the uterine epithelium, expression increased significantly for GH and IGF-2, reaching a peak at days 24 and 21 of pregnancy, respectively. The increases in GH-R expression after attachment were not significant, although higher than those observed during the final two days of pregnancy (Figure 1). PRL expression declined over all the stages analyzed, but there were no significant differences in expression from day 18 to day 25 of pregnancy (Figure 1).

## Discussion

The tammar placenta produces the pituitary hormones *GH *(and its receptor), *PRL *and *LH-β*. Until recently, marsupial placentas were thought to produce only limited hormones, but their presence at least in the tammar suggests that these endocrine factors may be intrinsic to normal pregnancy and preparation for lactation in all mammals. These results also extend previous data indicating an important role for IGF2 in the marsupial placenta.

There is now considerable evidence in both sub-classes of therian mammals (the eutherians and the marsupials) that the feto-placental unit produces local and systemic effects on the reproductive tract that result in a maternal recognition of pregnancy [17-20]. In macropodid marsupials, these responses include increases in size and secretory activity of the gravid versus non-gravid endometrium, as a direct result of the presence of a developing embryo [20-23]. It was hypothesized that the fetal effect may be due to either an endocrine factor or to an inflammatory effect [17,20]. This embryonic signal may be LH-β, or more correctly, a chorionic gonadotrophin as the placental specific product is designated in eutherian mammals. Preliminary evidence for a bioassayable chorionic gonadotrophin was obtained with a bioassay (MB Renfree and L. Wide, unpublished results) but this was never investigated further.

**Figure 4:**
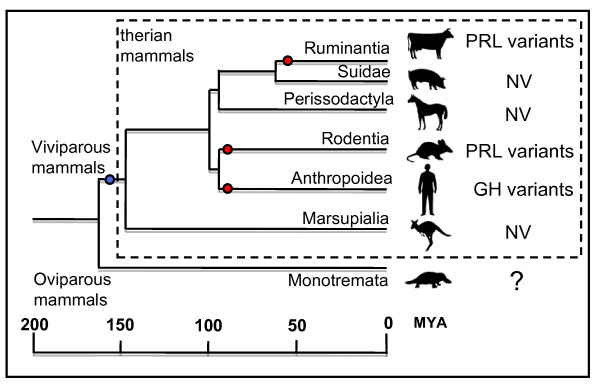
**Origin of pituitary hormone expression in the mammalian placenta**. Expression of the pituitary hormones GH, GH-R, PRL and LH-β in the yolk-sac placenta of the tammar wallaby suggest that these hormones may have provided a general function in the placenta of the common ancestor of therian mammals (blue dot). From this generalized expression, the placentas of some eutherian mammals including humans have developed specific gene copies including HCG, GH-V (red dots; NV indicates no sequence variants in those species; GH, growth hormone; GH-R, growth hormone receptor; GH-V, growth hormone variant; HCG, human chorionic gonadotrophin; LH-β, luteinizing hormone subunit-β; PRL, prolactin; NV, no variants).

The present study confirms that LH-β is synthesised by the placenta, and suggests that this, together with GH and PRL, may be responsible for the observed stimulation of the uterine endometrium to maintain pregnancy. It also establishes that these pituitary hormones may be a shared ancestral feature of the therian mammalian placenta (Figure 4).

PRL and LH*-β *receptors are present in the brushtail possum ovary [24], and PRL receptors are under strict regulation during pregnancy in the tammar wallaby corpus luteum and mammary gland [25]. However, there is a prolactin pulse about eight hours before parturition in the tammar which is essential for the establishment of lactation [26,27], but this cannot derive from the placenta because placental PRL mRNA is at its lowest before birth. Placental PRL can stimulate the endometrium to support the embryo and fetus in domesticated species such as the sheep [28]. However, given the low levels of expression in the tammar placenta, absence of significant quantitative changes during attachment and low levels of PRL receptor expression in the endometrium relative to the corpus luteum (H. Clark personal communication) it is unlikely to have a similar function in the tammar.

To date, only a few endocrine signals from the marsupial placenta have been detected, including prostaglandin F2α, relaxin, insulin and IGF-2 [29-32], as well as incipient steroid production [33-35]. The isolation of *GH*, GH-R, *PRL *and *LH-β *from the placenta of a marsupial shows that it is a much more complex organ than initially thought. The placental tissues in this study were examined between days 18 to 26 of pregnancy, the period between the first attachment of the yolk-sac placenta (day 18 of pregnancy) and full term (birth: day 26 to 27) when this tissue is likely to be most metabolically active. Before this period (that is, blastocyst to late vesicle stage), there is no evidence that the embryo provides a signal to the uterus as it is surrounded by a shell coat produced by the oviducal and uterine epithelial cells, and both uteri respond to the increased circulating progesterone to stimulate the uterine epithelium to become secretory [20,21,36,37]. Once the shell coat ruptures, the embryo makes a close interdigitation with the uterine epithelium and maternal blood supply. The fetus develops rapidly and organogenesis is complete by the time of birth only nine days later [10,38]. IGF-2 and GH are good candidates to facilitate this rapid growth.

IGF-2 is maternally imprinted in the tammar placenta, similar to all other eutherian species so far investigated [39]. The presence of genomic imprinting suggests that IGF-2 plays a dominant role in the sequestration of maternal resources for the fetus. However, the up-regulation of other important growth genes such as GH and its receptor suggests that the marsupial placenta is not simply an inert tissue capable of transferring uterine secretions to the fetus but, like that of eutherian mammals, must actively grow and secrete critical hormones so it can nourish the continued growth of the conceptus.

## Conclusions

The discovery of somatotropins and gonadotropins in the yolk-sac placenta of the tammar wallaby supports the growing knowledge about marsupial placental function and demonstrates that the marsupial placenta is a fully functional and complex organ of physiological exchange between mother and fetus. It also suggests an ancestral role for these hormones in the reproduction of all mammals.

## Methods

### Animals

Tammar wallabies of Kangaroo Island origin were maintained in our breeding colony in large grassy outdoor enclosures. Their grass diet was supplemented with lucerne cubes and vegetables while drinking water was provided *ad libitum*. All experimentation was approved by the University of Melbourne Institutional Animal Ethics Committees and conformed to the Australian National Health and Medical Research council (2004) guidelines.

### Tissue collection

Whole placentas were taken opportunistically from fetuses collected for other experiments. Both bilaminar and trilaminar regions of the placenta were carefully dissected away from the fetus and endometrium and placed into RNA-free cryotubes, snap-frozen in liquid nitrogen and stored at -80°C.

### RNA extraction and gene cloning

Total RNA was extracted from 50 mg to 200 mg of placental tissue using RNAwiz (Ambion, Austin USA) {AU Query: Please add city in Texas and USA} according to the manufacturer's protocol. RNA quality was assessed by electrophoresis on ethidium bromide gel and detection of clean 18 and 24 S bands. Intact samples were DNAse treated using a DNAfree kit (Ambion, Austin USA) {AU Query: the city and USA should be inserted here, too} and stored in RNA secure water at -80°C. RNA concentrations were determined using a NanoDrop Spectrophotometer (Thermo Scientific, Wilmington USA) {AU Query: Please add the city, state and country for Thermo Scientific} after which precisely 1 μg of total RNA was treated using the Superscript III First Strand Synthesis System for RT-PCR (Invitrogen, Carlsbad USA) {AU Q} uery: Please add the city and state for Intitrogen} to generate complimentary DNA (cDNA). Full length sequences were obtained by reverse transcriptase PCR using placental cDNA, GoTaq (Promega, Madison USA) {AU Query: Please add the city, state and country for Promega} master mix and primers from conserved regions of the brush-tailed possum sequences (*GH*: AF052192; *PRL*: AF054634; *LH-β*: AF017448; *GH-R*:AF467545). The specificity of these transcripts was then confirmed by BLAST homology and identification in the tammar wallaby trace archives in NCBI (GenBank accession numbers: *GH*: EU918392; *GH-R*: EU682376; *LH-β: *FJ434244; *PRL*: FJ434245). {AY Query: Need to add the GenBank accession numbers} Species specific primers were then designed from the full length *GH *sequence for use in qPCR analysis. QPCR primers for IGF-2 were replicated from Ager *et al *(2008) [31]. Primers were designed to span introns so that only cDNA was detected as confirmed by gel electrophoresis. QPCR was performed using the Quantitec Sybr Green PCR Kit (Qiagen, Germantown USA) {AU Query: Please add the city, state, and country for Qiagen} and reactions run in triplicate on an Opticon 2 Monitor (MJ Research, Waltham USA) {AU Query: Please add the city, state, and country for MJ Research}. The quantity of transcripts from the genes of interest were compared to the housekeeping gene β-actin Fwd, TTGCTGACAGGATGCAGAAG, Rev, AAAGCCATGCCAATCTCATC by comparison of amplification thresholds using Opticon 2 Monitor software. All primers were purchased from Sigma-Aldrich and qPCR products confirmed by BLAST using the least stringent parameters in NCBI. Transcripts were sequenced at the Gandel Charitable Trust Sequencing Centre (Monash University). Primer sequences, annealing temperatures and PCR cycle lengths can be found in Table 2.

The protein sequences of LHβ and PRL are provided in Additional file 1, Figure S1 and Additional file 2, Figure S2. The sequence of GH [40], GH-R [5] and IGF2 [38] were previously published.

### Statistics

Expression analysis of the pituitary hormones GH, GH-R and LH-β by standard PCR were repeated in BYS and TYS samples from three different individuals at days 18, 21, 23, 24 and 25 of pregnancy. Quantitative analysis was performed on TYS samples (Four to six per stage) also from days 18, 21, 23, 24 and 25 of pregnancy. Analysis of variance and multiple comparison tests were performed using Systat Version 13 and statistically significant outcomes reported as *P *< 0.05. Data are presented as means ± s.e.m.

## List of abbreviations

BYS: bilaminar yolk-sac; GH: growth hormone; GH-R: growth hormone receptor; IGF-2: insulin-like growth factor-2; LH-β: luteinizing hormone subunit-β; PRL: prolactin; TYS: trilaminar yolk-sac.

## Competing interests

The authors declare that they have no competing interests.

## Authors' contributions

MBR and BRM conceived the experiments. MBR collected and dissected the tissues. BRM performed the molecular analyses. AJP assisted with the analyses. All authors contributed to the intellectual design of the experiments and the writing of the manuscript. All authors read and approved the final manuscript.

## Supplementary Material

Additional file 1 **Figure S1: Alignment of the predicted protein sequence for tammar prolactin**. The PRL precursor was highly conserved between vertebrate species including the six cysteine residues (indicated by the red letters) at positions 33, 40, 87, 203, 220, and 228 relative to the wallaby. These amino acids form three disulfide bonds contributing to the three-dimensional structure of the mature protein. There are 14 amino acids (identified by asterisks) in mammals which are necessary for GH receptor binding site 1. All of these amino acids are conserved in the tammar. Additionally, Ala51 and Gly158 (indicated by the black arrows) are necessary for binding at site 2 and these are also conserved in the tammar and other vertebrates. Click here for file

Additional file 2 **Figure S2: Predicted protein sequence for tammar luteinizing hormone β subunit**. The protein sequences for both sub units (α and β) have already been described for the red kangaroo [15]. The predicted tammar sequence was identical to the red kangaroo except for an additional leucine at position 14 relative to the tammar. Click here for file
