# Supplementary figures and images for: Placental expression of pituitary hormones is an ancestral feature of therian mammals
Source: EvoDevo. 2011 Aug 19;2:16. doi: 10.1186/2041-9139-2-16 (PMC3170617; doi:10.1186/2041-9139-2-16)

## Slide 1
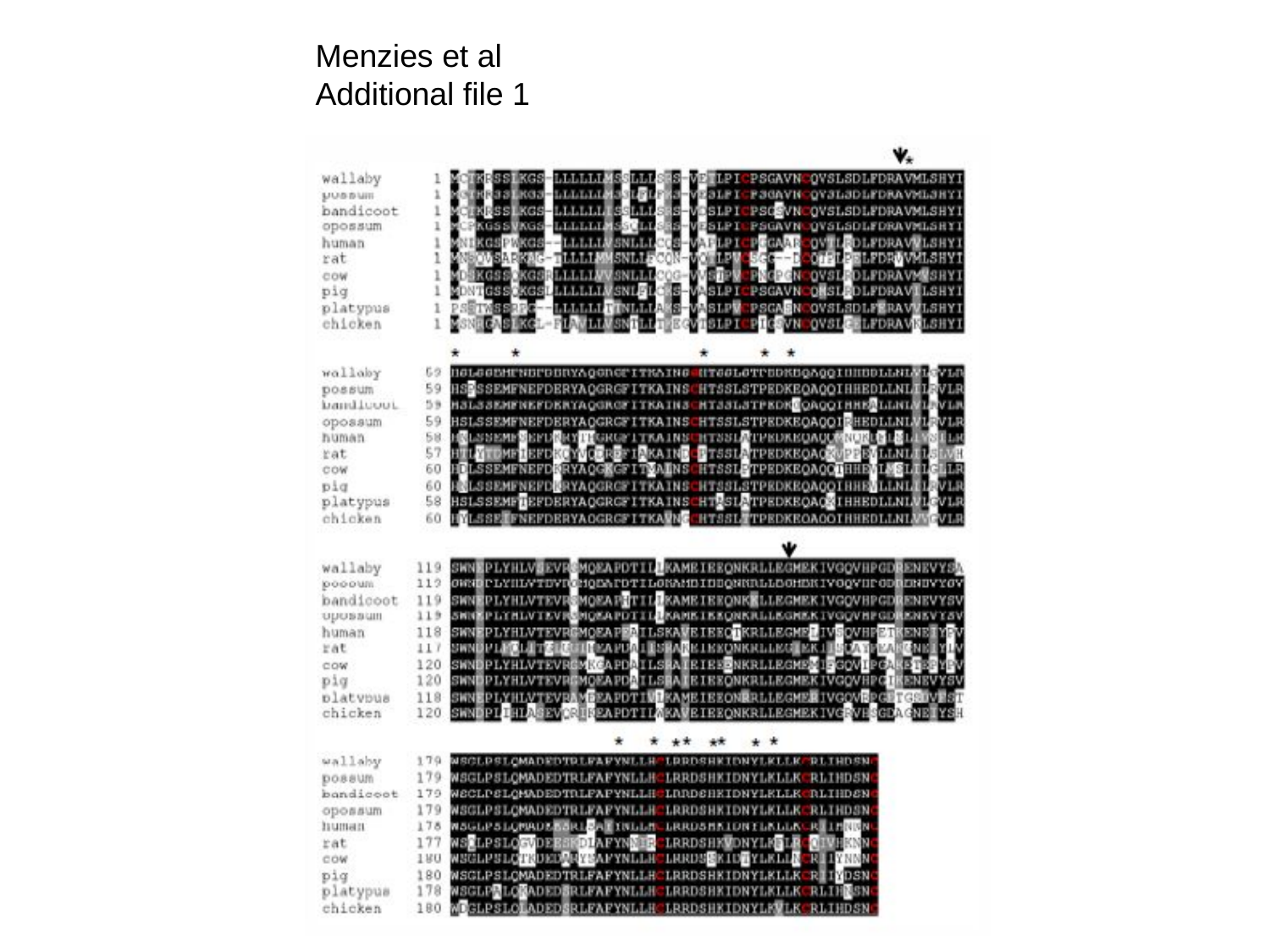

Menzies et al
Additional file 1

Supplement: Additional file 1 — Figure S1: Alignment of the predicted protein sequence for tammar prolactin. The PRL precursor was highly conserved between vertebrate species including the six cysteine residues (indicated by the red letters) at positions 33, 40, 87, 203, 220, and 228 relative to the wallaby. These amino acids form three disulfide bonds contributing to the three-dimensional structure of the mature protein. There are 14 amino acids (identified by asterisks) in mammals which are necessary for GH receptor binding site 1. All of these amino acids are conserved in the tammar. Additionally, Ala51 and Gly158 (indicated by the black arrows) are necessary for binding at site 2 and these are also conserved in the tammar and other vertebrates. [file 2041-9139-2-16-S1.PPT]

## Slide 1
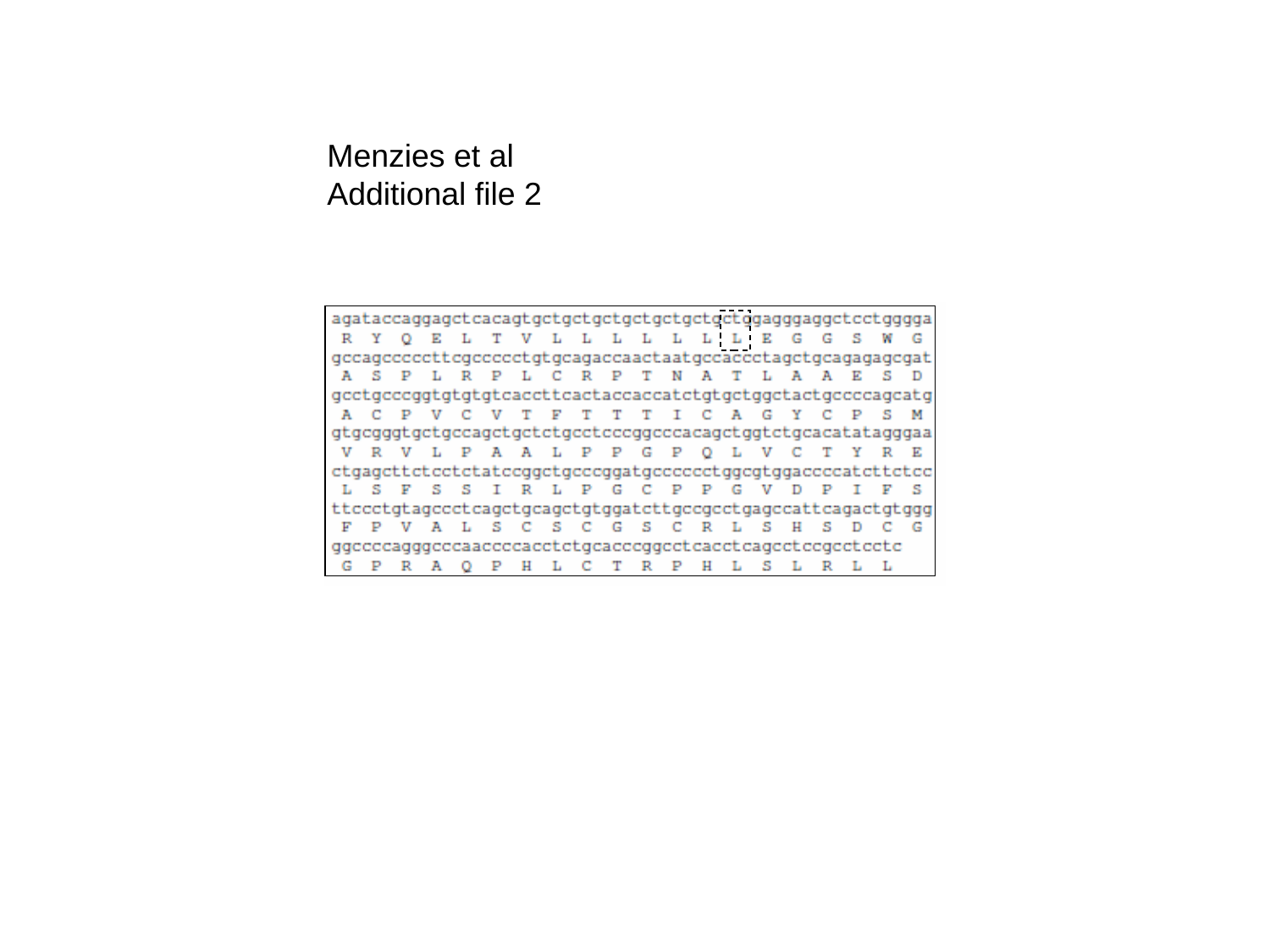

Menzies et al
Additional file 2

Supplement: Additional file 2 — Figure S2: Predicted protein sequence for tammar luteinizing hormone β subunit. The protein sequences for both sub units (α and β) have already been described for the red kangaroo [15]. The predicted tammar sequence was identical to the red kangaroo except for an additional leucine at position 14 relative to the tammar. [file 2041-9139-2-16-S2.PPT]
